# Comparison of finger flexor resistance training, with and without blood flow restriction, on perceptional and physiological responses in advanced climbers

**DOI:** 10.1038/s41598-023-30499-x

**Published:** 2023-02-25

**Authors:** Vidar Andersen, Espen Hermans, Vegard Vereide, Nicolay Stien, Gøran Paulsen, Jiří Baláš, Michail Lubomirov Michailov, Helene Pedersen, Atle Hole Saeterbakken

**Affiliations:** 1 Faculty of Education, Arts and Sports, Western Norway University of Applied Sciences, 133 6851 Sogndal, PB Norway; 2 Department of Physical Performance, Norwegian School of Sport Sciences, Oslo, Norway; 3 Faculty of Physical Education and Sport, Charles University, Prague, Czechia; 4 Department Theory and Methodology of Sports Training, National Sports Academy, Sofia, Bulgaria

**Keywords:** Psychology, Muscle

## Abstract

This study compared perceptional and physiological responses of finger flexor exercise performed with free flow and blood flow restriction (BFR). Thirteen male advanced climbers completed three sessions of finger flexor resistance exercise at (1) 40% of MVC (Low) and (2) 75% of MVC (High) and (3) BFR at 40% of MVC (Low + BFR) in a randomized and counterbalanced order. Rate of perceived exertion for effort (RPE) and discomfort (RPD), session pleasure/displeasure (sPDF), exercise enjoyment (EES), lactate concentration and oxygen saturation were recorded after the last set. Both low-intensity sessions induced higher RPD than High (p = 0.018–0.022, ES = 1.01–1.09) and High was perceived as more enjoyable than Low-BFR (p = 0.031, ES = 1.08). No differences were found for RPE or sPDF (p = 0.132–0.804). Lactate was elevated more after High than the Low-sessions (p < 0.001, ES = 1.88–2.08). Capillary oxygen saturation was lower after Low + BFR compared to the other sessions (p = 0.031, ES = 1.04–1.27). Finally, the exercise volume was greater in Low compared to High (p = 0.022, ES = 1.14) and Low + BFR (p = 0.020, ES = 0.77). In conclusion, among advanced male climbers, performing Low + BFR led to a similar exercise volume but was perceived as more discomforting and less enjoyable compared to High. The Low session yielded similar responses as the Low + BFR but required a much greater exercise volume.

## Introduction

During the last decades, sport climbing has gained increased interest among both practitioners and scientists. Climbing performance depends on multiple components such as anthropometric-, physiological-, psychological- and technical factors^1–3^. Furthermore, finger flexor strength has been considered as one of the most important determinants of the physiological factors (e.g., body composition, muscle hypertrophy, flexibility and aerobic and anaerobic capacity) to predict climbing performance^1^. In a recent meta-analysis, Stien et al.^4^ reported that specific resistance training of the finger flexors increases finger strength and performance in climbing specific tests, when compared to just climbing training. Therefore, specific resistance training of the finger flexors can be recommended to improve factors related to climbing performance and consequently may, in addition to climbing, be a viable strategy to increase climbing performance. At the same time climbers report injuries in the finger/hand as the injury with the highest prevalence^5,6^. Consequently, increasing the total strain on the fingers by adding specific finger training could also increase the risk of being injured.

Blood flow restriction (BFR) training has shown comparable chronic effects in hypertrophy and strength as traditional heavy resistance training^7,8^. In BFR training a pneumatic cuff is used to apply pressure around the limbs, partially restricting arterial blood flow and occluding the venous return. The BFR leads to a reduced oxygen saturation in muscles distal to the cuff during both exercise and rest intervals^9^, which accelerates the accumulation of metabolites and limits the recovery process during rest intervals, compared to the free flow condition^10^.

Importantly, the mechanical load normally used in BFR training is considerably lower (i.e. 20–50% of 1 repetition maximum (RM)) compared to heavy resistance training (70–85% of 1-RM)^11^. Further, the same increases in strength may also be achievable by conducting resistance training with low intensity without BFR, however, this would require a substantial increase in the training volume^12^. Notably, although low intensity (with and without BFR) have been shown to increase maximal strength, several studies report heavy resistance training to be more favorable^13–15^. However, considering the potent effects of BFR on hypertrophy and strength, BFR training of the finger flexors could be a viable alternative for climbers when conducting specific resistance training of the finger flexors. Compared to heavy resistance training, BFR will purposely lower the mechanical strain on the finger flexors and finger joints, due to reduced load, and may therefore reduce the risk of injuries^16^.

The willingness to continue with a physical activity and training likely depends on how the activity is perceived^17,18^. Traditionally, BFR training is performed until fatigue^19^. Hence, it may be perceived just as or even more exertive and discomforting than traditional resistance training performed with heavy or light loads. Further, increased discomfort, could affect how the session is experienced regarding session displeasure/pleasure and enjoyment. Due to the different definitions and interpretations of the rate of perceived exertion (RPE), it has been recommended to accompany RPE with other affective measures such as rate of perceived discomfort (RPD)^20^. A recent meta-analysis, consisting of 30 studies, examined both effort and discomfort when analyzing the acute effects of blood flow restriction training^21^. The authors reported similar RPE for BFR and non-BFR when performing sets to voluntary failure, while RPD appears to be higher for BFR compared to high load non-BFR training^21^. For example, Bell et al.^22^ compared low intensity BFR training with different cuff pressures with free flow low and high intensity resistance training among resistance-trained individuals. All conditions were performed until failure. They reported that BFR led to greater discomfort than free flow, however, effort was only increased when a higher cuff pressure was implemented. Miller et al.^23^ compared low intensity BFR training with either low or high intensity without BFR when completing a matched training volume. Unlike Bell et al.^22^, the greatest perception of effort was reported with free flow high intensity training, whereas low intensity with BFR was perceived more strenuous than low intensity without BFR. Furthermore, free flow high intensity training and low intensity BFR training led to more discomfort than free flow low intensity training. Inconsistent with these findings, when comparing resistance training with low and high intensity without blood flow restriction, low intensity resistance training has been reported as the more discomforting condition^12^.

To our knowledge, there have been no studies comparing the acute effects of climbing specific finger flexor resistance training conducted with BFR against low and heavy load free flow training. It would be of great practical and scientific interest to examine how the different conditions influence training volume, physiological responses (e.g. lactate and oxygen saturation) and different affective measures, such as effort, discomfort, session displeasure/pleasure and exercise enjoyment among active advanced level climbers. Therefore, the aim of this study was to compare the acute affective, physiological and training volume effects of conducting finger flexor training with either high intensity (High), low intensity with BFR (Low + BFR) or low intensity without BFR (Low). We hypothesized that conducting Low + BFR would lead to (i) a reduced exercise volume compared to Low, but similar to High, (ii) greater perception of discomfort compared to training without BFR, (iii) being less enjoyable and more displeasing than training without BFR, and (iv) lower oxygen saturation but greater lactate accumulation compared to training without BFR. Further, we hypothesized no difference in perception of effort between the sessions, but that the Low would be more discomforting than the High session.

## Results

**Figure 1:**
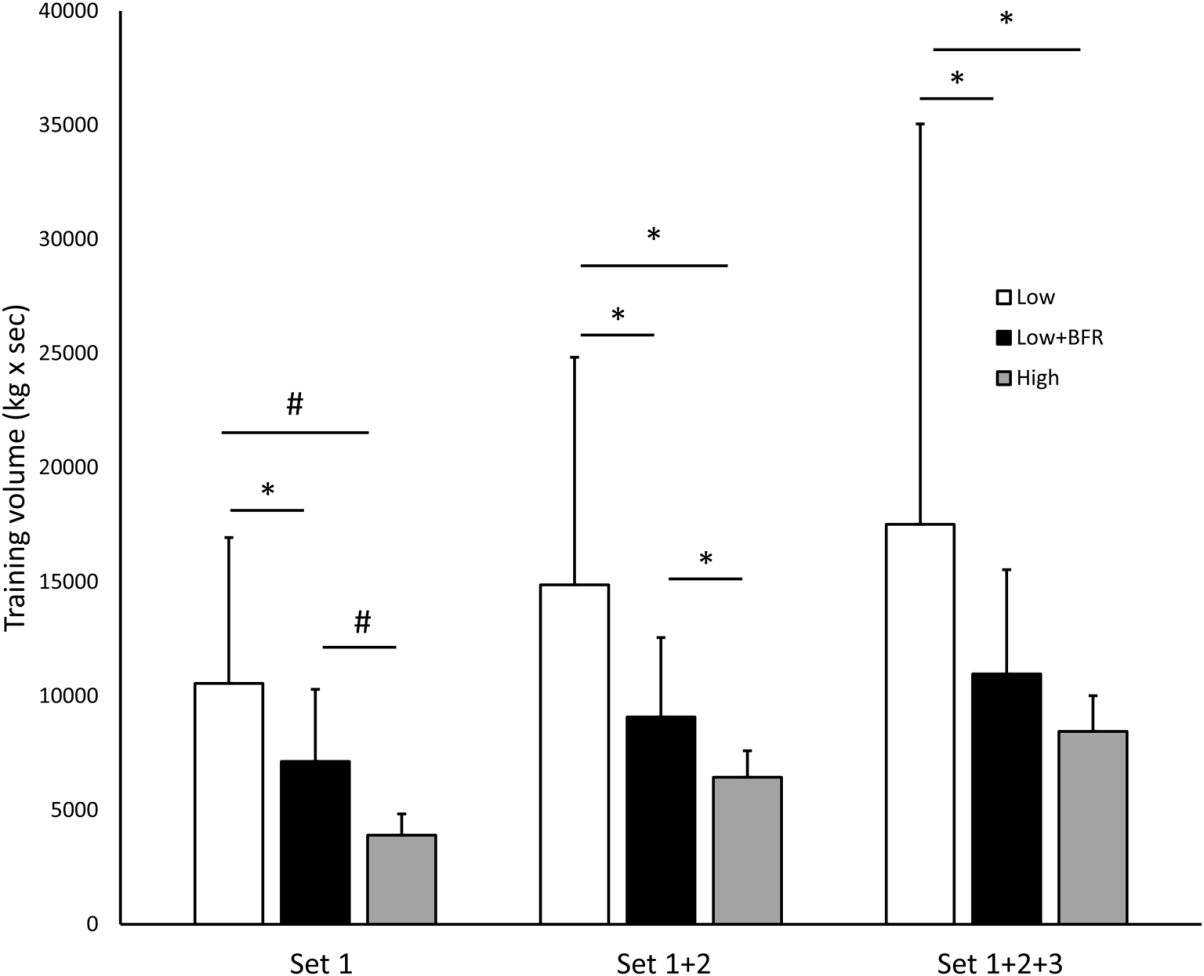
Accumulated training volume (kg x sec) in set 1, set 1 + 2, and set 1 + 2 + 3 in the three sessions Low, Low + BFR, and High. Data presented as mean and standard deviation. *p < 0.05, #p < 0.01.

The total exercise volume was higher in the Low when compared to the Low + BFR (60%, p = 0.020, d = 0.91, Fig. 1) and the High session (107%, p = 0.022, d = 0.90). There was no statistical difference between the Low + BFR and High (p = 0.066, d = 0.73). The summated volume after the first and the second set showed a similar pattern with Low being higher than Low + BFR (48% and 64%, p = 0.025 and 0.037, d = 0.88 and 0.81) and High (171% and 131%, p = 0.006 and = 0.022, d = 1.08 and 0.89). Further, the exercise volume achieved in Low + BFR was higher than in the High session (83% and 41%, p = 0.007 and = 0.016, d = 1.10 and 0.94).

For the perception variables the Friedman test showed a difference between the conditions in the RPD (p = 0.037, Table 1) and EES (p = 0.009), but not in the RPE (p = 0.804) and sPDF (p = 0.132). Comparing the different conditions, the RPD was in average higher for both the Low (2 points, p = 0.022, r = 0.63) and the Low + BFR (3 points, p = 0.018, r = 0.66) when compared to High. There was no statistical difference between Low and Low + BFR (p = 0.527, r = 0.18). For the EES there were no statistical difference between the Low and the two other conditions (Low + BFR; p = 0.051, r = 0.54 and High; p = 0.399, r = 0.23), however, a statistical difference of 2 points on the EES scale was observed between Low + BFR and High (p = 0.031, r = 0.60).

**Table 1:** Perceptive (median ± interquartile range) and physiological (mean ± SD) measures for the different sessions.

	Low	Low + BFR	High
RPE (0–10)	10 ± 1	10 ± 1	9 ± 1
RPD (0–10)	8 ± 2	9 ± 1	6 ± 3^A,B^
sPDF (− 5–5)	4 ± 2	4 ± 2	4 ± 2
EES (1–7)	5 ± 2	3 ± 2	5 ± 2^B^
Lactate (mmol/L)	6.5 ± 1.9	6.0 ± 2.7	10.1 ± 1.7^A,B^
StO_2_ (%)	97 ± 1^B^	93 ± 4	96 ± 2^B^

*RPE* rate of perceived exertion effort, *RPD* rate of perceived exertion discomfort, *sPDF* session pleasure/displeasure, *EES* exercise enjoyment, *StO*_*2*_ oxygen saturation, *SD* standard deviation.

^A^Significantly different than Low (p < 0.05).

^B^Significant different than Low + BFR (p < 0.05).

The lactate concentration was significantly higher after the High session when compared to both Low (57%, p < 0.001, d = 2.23, Table 1) and Low + BFR (70%, p < 0.001, d = 1.67). There was no statistical difference between Low and Low + BFR (p = 1.000, d = 0.18). The StO_2_ during the Low + BFR session was significantly lower than the two other sessions (Low; 3%, p = 0.031, d = 0.84 and High; 3%, p = 0.031, d = 0.74). There was no statistical difference between Low and High (p = 1.000, d = 0.28).

## Discussion

In accordance with our hypothesis, the results of the present study showed that the Low session led to a greater exercise volume compared to the Low + BFR and High sessions. Further, as expected, BFR training led to lower oxygen saturation than both High and Low and was perceived as less enjoyable than the High session. Of note, there was a tendency of Low + BFR also being perceived less enjoyable compared to the Low session, however the difference did not reach statistical significance (p = 0.051). Also, in accordance with our hypothesis, the results showed that performing finger flexor resistance training with low intensity (with and without BFR) until failure led to greater levels of discomfort compared to high intensity without BFR. Finally, as expected, there was no difference in perceived effort between the three sessions. However, and in contrast to our hypothesis, the high intensity sessions led to greater levels of lactate concentration compared to the other sessions and there was no difference in perceived session pleasure between the sessions.

The difference in training volume between the two low intensity sessions is likely explained by an impaired recovery during the rest intervals both within and between the sets in the BFR session^19^. Restricting the blood flow will induce hypoxia in the muscle, which likely reduces the replenishing of energy storages during the rest intervals. This explanation is supported by our findings for arterial oxygen saturation showing that the BFR session had a reduction in saturation levels.

The lack of difference in exercise volume between Low + BFR and High was accompanied by oxygen saturation and lactate pointing in different directions. Due to the lower levels of oxygen saturation after Low + BFR, it was at first glance surprising to observe that the High session led to the highest accumulation of lactate. However, a higher load and the same volume (time) means the energy turnover was higher at High than Low + BFR and lactate is directly related to the glycolytic rate^24^. Thus, training load and energy demand were more important for the lactate response than the oxygen saturation. This explanation is supported by previous studies^25,26^. For example, Freitas et al.^26^ reported a higher lactate concentration after performing leg press and knee extension at 80% of 1-RM without occlusion when compared to 30% of 1-RM both with and without occlusion.

In the present study, the training volume in the BFR session was greater compared to the high intensity session after the two first sets, but not after the final set (although a statistical trend was observed). This could indicate that when multiple finger flexor sets are conducted using low intensity and BFR, the training volume progress to become relatively similar to the exercise volume using high intensity without BFR. This could be of great interest for climbers, since finger flexor strength has been identified as an important factor for performance^1^, but also one of the muscle groups with the highest prevalence of injuries^5^. Therefore, training these muscles with low intensity and relatively low volume using blood flow restriction, might be advantageous presumed that they yield the same strength adaptions.

The RPD findings showing greater rating for Low + BFR than High are in line with previous studies reporting that conducting BFR training leads to an increased level of discomfort/pain compared to free flow^22,27,28^, which is most likely a result of a higher accumulation of different metabolites during the BFR session^29,30^. However, the similar responses in discomfort between the two low intensities stands in contrast to previous studies^22,28^ reporting that low intensity with BFR yield higher discomfort than low intensity with free flow. Importantly, in the present study the training consisted of isometric contractions and focusing on smaller muscles, unlike the previous studies which used dynamic contractions and exercising larger muscles^22,28^. It has been shown that occlusion occurs under isometric contractions at an intensity similar as the one used in the present study (40% of MVC)^31^. Therefore, the prolonged contraction time in the Low session might have had a larger occlusion effect than in the High session, consequently leading to an increased accumulation of metabolites and increased levels of discomfort.

The increased enjoyment after the High vs. the Low + BFR session could be explained by the difference in discomfort. However, based on this assumption, we would also expect a difference in EES between the Low and the High session, which was not observed. Instead, the difference in EES might be explained by the BFR per se. Although not reaching statistical significance, there seems to be a meaningful difference also between the two Low-sessions (p = 0.051, ES = 0.67). Importantly, none of the participants were accustomed to performing BFR-training. Although they did conduct a familiarization session, it may not be sufficient to familiarize with wearing the inflated cuff. Therefore, having inflated cuffs on their upperarm may have affected the participants` experience of the training, making it less enjoyable. Our finding is in accordance with Suga et al.^32^ which compared light resistance training with and without BFR among young adults. They measured enjoyment using the PACES scale and found lower levels of enjoyment after the occlusion session.

The similarities in RPE were as expected and can be explained by the fact that all sets in all three sessions were performed until momentary failure. Hence the exertion is expected to be similar^33^. These findings, together with the studied populations, may also explain the lack of difference in session pleasure. Being active climbers at an advanced level exercising the finger flexors is most likely an important part of their training routine. Since all sessions were perceived as similarly exertive, it seems likely that they would be rated as similarly good/bad on the sPDF scale. Notably, it has been reported that the feeling of working hard is important for the preference of a workout^34^.

The study has several limitations. First, the participants in the study were active male climbers at an advanced level and the findings can not necessarily be generalized to other populations. Second, the rest intervals between the sets in the high intensity and the two intensity sessions were different, which may have affected the results. Also, the training volume was not equated and consequently the effect of the BFR cannot be isolated. Importantly, the design is similar to previous studies^22,35^ and was chosen to imitate how these sessions are performed in regular training. Also, it was of importance to follow previous recommendations and keep the total time of the occlusion session in the range of ten to twenty minutes^19^. Further, although the scales were introduced to the participants in a familiarization session, they had no prior experience with them. Therefore, the rating may have been different if they have had more experience with the scales. Importantly, the order of the sessions was randomized to prevent bias from a potential learning effect. Finally, the measurements were only collected after the final set of each session. Therefore, we may have missed interesting changes in perception and physiological parameters throughout the sessions. Of note, the aim of the study was to compare the measures between the complete sessions and not to observe potential changes throughout the sessions.

In conclusion, performing a finger flexor resistance training session with low intensity and BFR led to a similar exercise volume but was perceived as more discomforting and less enjoyable than the high intensity session in advanced male climbers. Performing the session with low intensity without BFR yielded similar affective responses as the low intensity with BFR but required a much greater exercise volume. These findings suggest that active climbers who want to reduce the mechanical stress on the fingers should prioritize low intensity BFR-training for the specific finger flexor resistance training. However, one should be aware that this training may be perceived as more discomforting and less enjoyable than the more traditional high intensity resistance exercise. Future studies should compare the long-term effects of finger flexor resistance training performed with either low intensity and BFR or high intensity without BFR in climbers.

## Methods

### Experimental design

**Figure 2:**
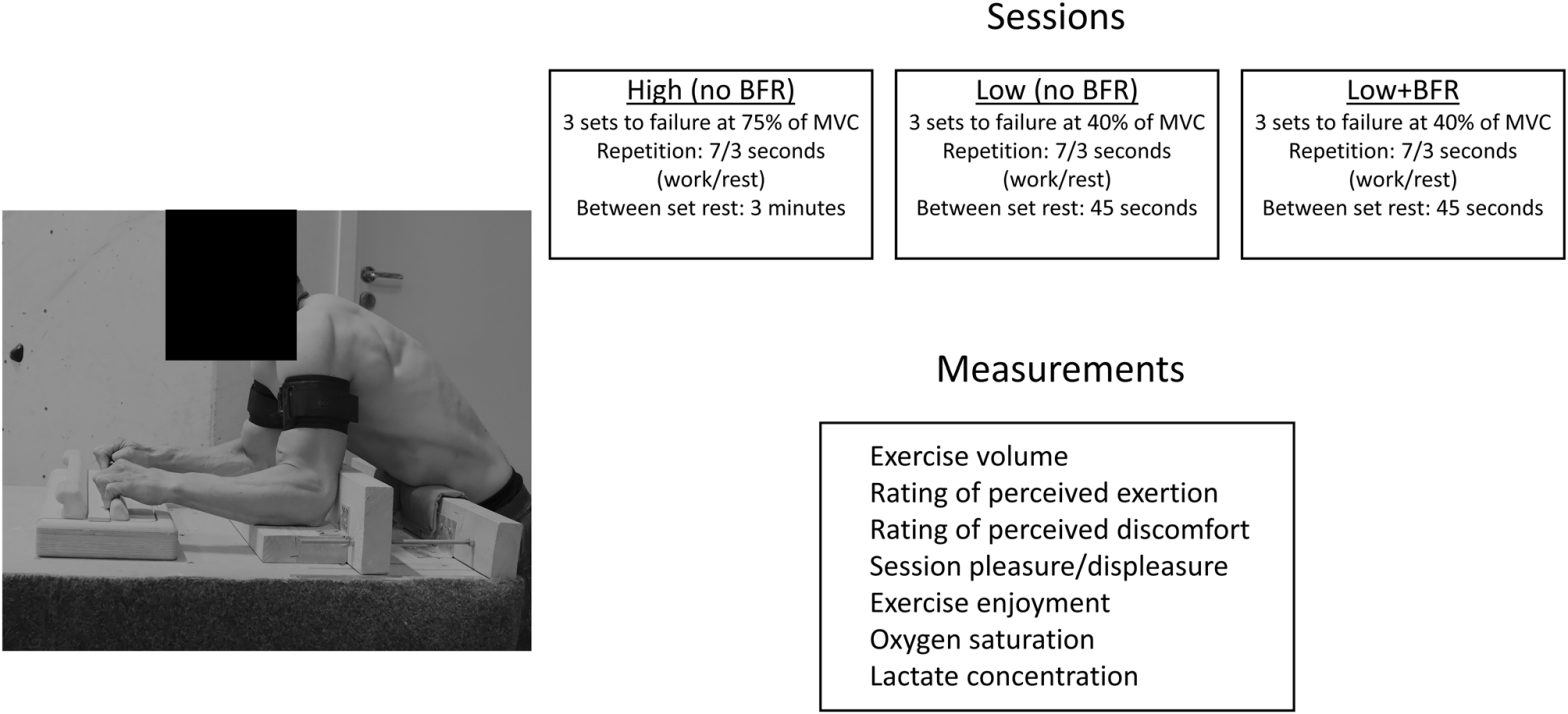
Overview of the study design and finger flexor training apparatus.

A within-subject crossover design was used to compare the acute physiological and affective responses when performing resistance training of the finger flexors with either High, Low, or Low + BFR. In total, the participants had one familiarization session and three experimental sessions in the lab. For each individual, all sessions were scheduled at the same time of day with a minimum of 48 and a maximum of 96 h separating each session. The order of the experimental sessions was randomized and counterbalanced. The finger flexor training consisted of three sets performing intermittent, isometric contractions (work-rest ratio of 7:3 s) to failure using either High (75% of MVC), Low + BFR (40% of MVC + BFR) or Low (40% of MVC). Exercise volume was recorded during the sessions. Immediately after completing the final set the lactate concentration and oxygen saturation (StO_2_) were measured. Ten minutes after the session ended, the participants were asked how they perceived the session related to effort, discomfort, pleasure/displeasure and enjoyment. For an overview of the design see Fig. 2.

### Participants

Fifteen active climbers performing at an advanced level were recruited to participate in the study. The sample size was justified by performing an a priori power analysis in SPSS based on the difference in rating of perceived discomfort between the conditions reported in a previous study (mean ± SD; 7.1 ± 1.4 vs 5.0 ± 2.5)^23^, an alpha level of 0.05 and power of 0.8. Two of the participants dropped out due to factors not related to the project. The thirteen climbers (age; 27 ± 8 years, height; 178 ± 6 cm, body mass; 71 ± 7 kg) who completed all tests had an average of nine years of climbing experience (range 2–32 years) and a redpoint performance of 21 ± 3 (range 18–26) on the International Rock Climbing Research Association (IRCRA) scale. The inclusion criteria were active male climbers over 18 years of age, a minimum self-reported climbing level of 18 on the IRCRA scale and free of injuries that could limit their performance in the training sessions. The participants agreed to refrain from alcohol, resistance training of the finger flexors and climbing 48 h in advance of each test session. They were informed orally and in writing about the procedures. Informed consent was obtained from all participants before they were enrolled in the study. The study was conducted according to the University College’s ethical guidelines, approved by the Regional Committee for Medical and Research Health Ethics, South-East Norway (2018/1345) and assessed by the Norwegian Centre of Research Data (527474).

### Procedures

A familiarization session was conducted 2–5 days before the first experimental session. In this session the anthropometrics of the participants were measured, the training equipment was adjusted to the individual and these setting were noted, the participants tried the three different training modalities (two minutes per modality), and they were introduced to the different scales for affective measures.

The resistance training for the finger flexors was conducted on a custom-made apparatus (Fig. 2)^36,37^. A fingerboard (Climbro, Sofia, Bulgaria) with an embedded force sensor was attached to a table. Wooden boards were attached (to the table) with adjustable screw clamps to adjust the distance between the fingerboard and the elbows. The distance was standardized with a 90-degree angle in the elbow so that the participants could use a half-crimp grip with passive thumbs (both hands simultaneously). During the training, the arms had to rest on the apparatus. This secured that the force output was created by the finger flexors and not by tilting the body or using the back- or arm muscles. A 23 mm rung was used in all tests. Chalk was allowed to ensure good friction between the rung and the fingers. Also, the rung was brushed before each session. The fingerboard was connected via bluetooth to the mobile application which showed force, repetitions, and time in target zone.

Before each test session the participants followed the same warm-up protocol consisting of 10 min of easy, but progressive bouldering. The general warm-up was followed by a specific warm-up on a fingerboard, consisting of four contractions (50–80% of self-perceived maximum) lasting five seconds each. Afterwards, the participants performed two maximal voluntary contractions (MVC) on the apparatus, each lasting 5 s. A rest interval of 60 s separated the attempts. The MVC was performed to calculate the target zone-threshold of the different sessions and was conducted on each test day to account for day-to-day variability. Each training session consisted of three sets and started five minutes after the last MVC. Each set consisted of isometric, intermittent contractions that lasted for seven seconds and were separated by three seconds of rest. The arms and fingers were kept in position throughout the set. The participants were instructed to keep the force close to the set intensity, however, some fluctuations were allowed if they stayed within the target zone (defined as ± ten percent of the set intensity). Each set was performed until failure, being defined as two seconds of continuous force output below the lower limit of the target zone for that specific session. Only the completed repetitions were included in the calculation of training volume, i.e. the seconds of the uncompleted final repetition were excluded.

The design of the different training sessions was similar to other studies comparing heavy resistance training with blood flow restriction training at low intensities^35^. The high intensity session consisted of three sets of repetitions to failure at 75% of MVC. The rest intervals between the sets were three minutes. The two low intensity sessions (Low and Low + BFR) consisted of three sets of repetitions to failure at 40% of MVC. The rest intervals between the sets in these sessions were 45 s.

The blood flow restriction was applied using two inflatable cuffs (Occlude, Blood flow restriction exercise, Denmark, (width: 9 cm x length: 38 cm)). This brand of cuffs has shown good reliability both between sessions (ICC 0.86 and CV 5.7%) and rater (ICC 0.98 and CV 3.2%), when measuring occlusion pressure for the upper body while standing up^38^. The cuffs were placed as proximately as possible on each arm. To determine the pressure for each individual, the cuffs were progressively inflated while monitoring the pulse using a doppler-apparatus (SonoTrax Series Ultrasonic Pocket Doppler, Edan Diagnostics, San Diego, California, USA). The 100% occlusion pressure was defined as the pressure when the pulse was no longer noticeable. The cuffs were then deflated, before being inflated until 60% of the 100% occlusion pressure which is consistent with previous recommendations for resistance exercise^19,39^. These measurements were performed while standing up to imitate the exercising condition as closely as possible. The cuffs were not removed before the session was completed. However, after each set, the pressure was controlled and if necessary, adjusted before the next set.

### Measurements

The number of successfully completed repetitions were continuously noted throughout the sessions. Training volume was defined as the product of training duration (number of completed repetitions multiplied by 7 s) multiplied with the load for that specific session (in kg). Immediately after the final set was complete, StO_2_ and lactate concentration were measured. For the BFR condition these measurements were performed before removing the cuff. StO_2_ was measured using a pulse oximeter (Contec CMS 50D Pulse Oximeter, Qinhuangdao, China) placed on the left thumb. Lactate was measured using a Lactate Pro 2 analyzer (Arkray Inc, Kyoto, Japan). The right index finger was washed and cleaned before taking the blood sample. The first drop of blood was wiped off before sampling the blood.

The perception of the different sessions was measured using four different scales. The scales were presented to the participants ten minutes after the sessions and the participants were asked to consider the session in its entirety when replying to the questions. All scales were shown to the participants while the test leader read the question to them (also listed on top of the scales). The scales were translated from the original forms to Norwegian. Prior to the study, three of the authors (AHS, HP and VA) translated the forms independently before comparing, discussing, and agreeing on the final versions. These versions were then translated back to English by a professional. The new English versions were then compared with the originals. In general, there were only minor differences between the versions, which were adjusted after mutual agreement.

Based on previous recommendations, the perception of exertion was differentiated into effort and discomfort^40^. The rating of perceived exertion for effort scale (RPE) was used to measure effort, while the rating of perceived exertion for discomfort scale (RPD) was used to measure discomfort^33^. Both scales consist of 11-items and ranges from no effort/discomfort to maximal effort/discomfort. Based on previous recommendations^20^, the RPE scale was presented to the participants with the following phrase: “*How much of your perceived physical capacity out of your perceived maximum (where 10 being your maximum) did you invest to complete this workout?*”. The upper and lower values were anchored by the following sentence *“0 can be described as sitting still during the whole session while 10 would be maximal effort using your maximal physical capacity throughout the whole session”* The RPD scale was presented with the following phrase: “*Based on the completed session, how much discomfort did you feel, where 10 being where you could not imagine the sensations relating to physical activity being any more intense?*”^40^. The upper and lower limit were anchored by the following sentence *“0 can be described as feeling no noticeable sensation relating to the training while 10 would be the most intense training related sensation you could imagine”.*

How the participants perceived the session regarding pleasure/displeasure was measured using the session pleasure/displeasure feelings scale (sPDF). The scale is a bipolar 11-point scale stretching from − 5 (very bad) to 5 (very good), where 0 is considered neutral. The sPDF scale was presented with the following phrase: “*How was your workout*?”^41^ and the upper and lower limits were anchored by the following sentence *“− 5 can be described as perceiving the session as one of the worst/least pleasurable training sessions you have ever conducted while 5 would be one of the best/most pleasurable training sessions you have ever conducted”*. How much the participants enjoyed the sessions was measured using the exercise enjoyment scale (EES). The scale ranges from one to seven with one being “not at all” and seven being “extraordinary”. The scale was presented with the following question “*How much did you enjoy the exercise session?*”^42^. The upper and lower limits were anchored by the following sentence *“1 can be described as perceiving the session as one of the least enjoyable training sessions you have ever conducted while 7 would be one of the most enjoyable training sessions you have ever conducted”.*

### Statistical analyses

The statistical analyses were performed using SPSS (IBM Corp. Released 2020. IBM SPSS Statistics for Windows, Version 27.0. Armonk, NY: IBM Corp). For the non-parametric variables (RPE, RPD, sPDF and EES) the Friedman test was used to detect differences between the sessions. If differences were detected, the Wilcoxon signed rank test was used to identify where the differences lay. These results are presented as median ± interquartile range. For the parametric variables (Training volume, StO_2_ and lactate) normality was checked and confirmed by visual inspection. Differences between the sessions were assessed with one-way repeated measures ANOVAs with Bonferroni post hoc tests. These results are presented as mean ± standard deviation (SD). Effect size for the parametric variables is presented as Cohen`s d effect size (d) and was calculated using the following equation: mean session 1–mean session 2 divided by the standard deviations of the difference. An effect size of 0.2–0.5 was considered small, 0.5–0.8 medium and > 0.8 large^43^. For the non-parametric variables effect size was calculated as product-movement r (r) using the following equation: r = z/√n, with z being the z-value of the Wilcoxon signed ranked test and n being the number of participants. A product-movement r of 0.1–0.29 was considered small, 0.3–0.49 medium and ≥ 0.5 large^43^. Statistical difference was accepted at p < 0.05.

## Data Availability

The datasets analyzed during the current study are not publicly available due to the content of the agreement between the participants and responsible institution. However, they are available from the corresponding author on reasonable request.
