# Publisher Correction: Comparison of finger flexor resistance training, with and without blood flow restriction, on perceptional and physiological responses in advanced climbers

**DOI:** 10.1038/s41598-023-31266-8

**Published:** 2023-03-13

**Authors:** Vidar Andersen, Espen Hermans, Vegard Vereide, Nicolay Stien, Gøran Paulsen, Jiří Baláš, Michail Lubomirov Michailov, Helene Pedersen, Atle Hole Saeterbakken

**Affiliations:** 1 Faculty of Education, Arts and Sports, Western Norway University of Applied Sciences, 133 6851 Sogndal, PB Norway; 2 Department of Physical Performance, Norwegian School of Sport Sciences, Oslo, Norway; 3 Faculty of Physical Education and Sport, Charles University, Prague, Czechia; 4 Department Theory and Methodology of Sports Training, National Sports Academy, Sofia, Bulgaria

Correction to: *Scientific Reports* 10.1038/s41598-023-30499-x, published online 25 February 2023

The original version of this Article contained an error in the order of the Figures. Figures 1 and 2 were published as Figures 2 and 1. As a result, the Figure legends were incorrect.

**Figure 1:**
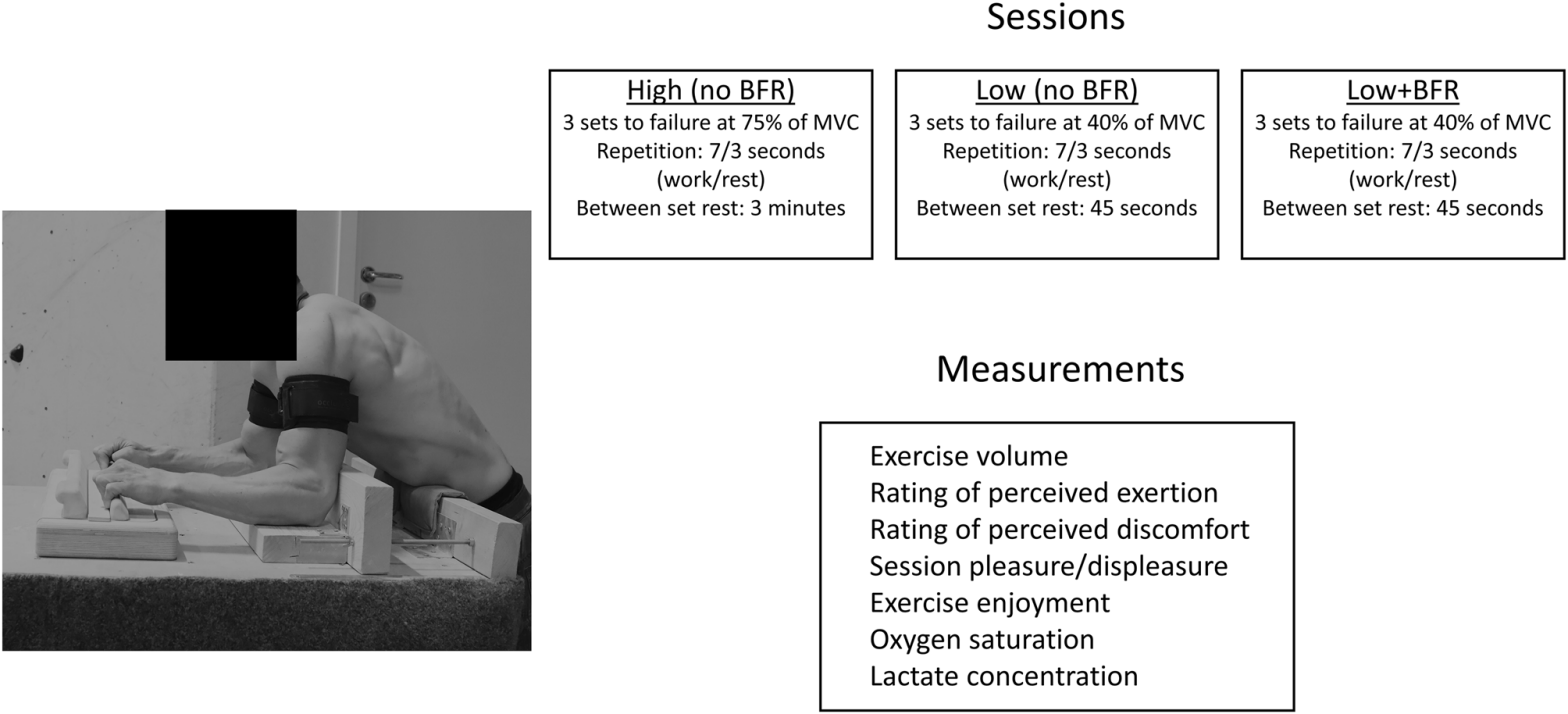
Accumulated training volume (kg × sec) in set 1, set 1 + 2, and set 1 + 2 + 3 in the three sessions Low, Low + BFR, and High. Data presented as mean and standard deviation. *p < 0.05, ^#^*p* < 0.01.

**Figure 2:**
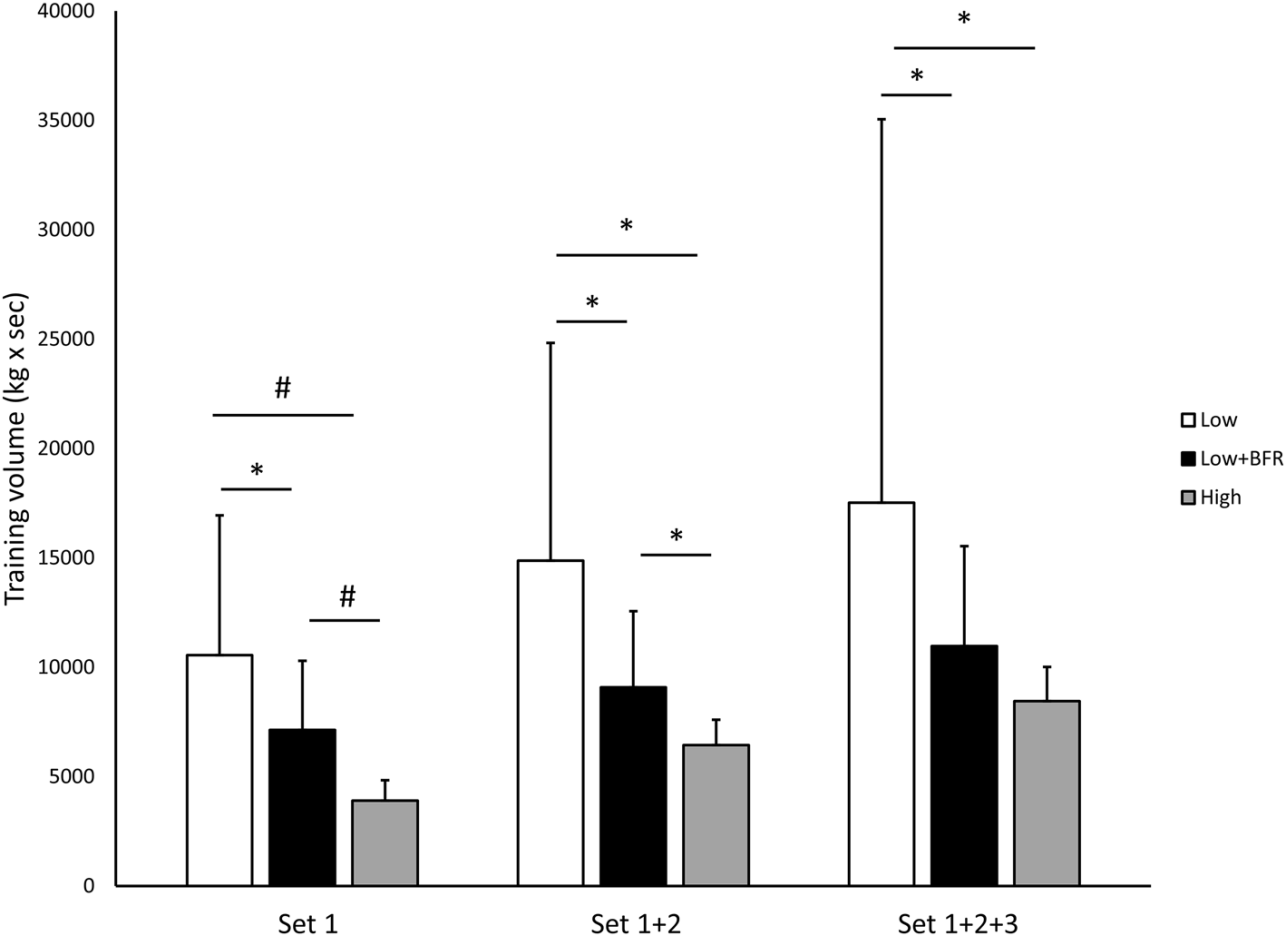
Overview of the study design and finger flexor training apparatus.

The original Figures 1 and 2 and accompanying legends appear below.

The original Article has been corrected.

